# Elucidation of the Glycan Structure of the b-type Flagellin of *Pseudomonas aeruginosa* PAO1

**DOI:** 10.1021/acsinfecdis.4c00896

**Published:** 2025-01-24

**Authors:** Paul J. Hensbergen, Loes van Huijkelom, Jordy van Angeren, Arnoud H. de Ru, Bart Claushuis, Peter A. van Veelen, Wiep Klaas Smits, Jeroen Corver

**Affiliations:** † Center for Proteomics and Metabolomics, Leiden University Medical Center, Leiden 2333 ZA, The Netherlands; ‡ Leiden University Center for Infectious Diseases, Leiden University Medical Center, Leiden 2333 ZA, The Netherlands

**Keywords:** Pseudomonas, glycosylation, mass spectrometry (MS), proteomics, cell motility, bacteria

## Abstract

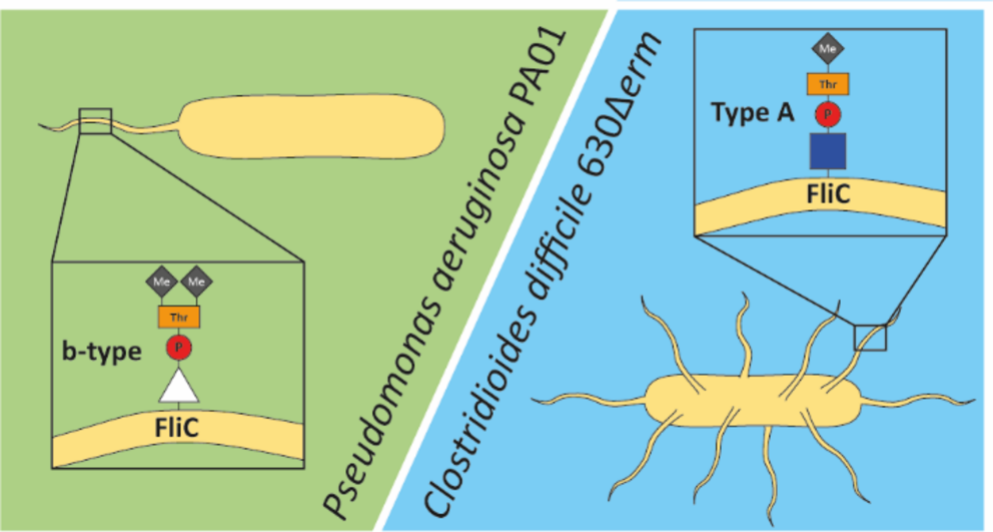

Flagella are essential for motility and pathogenicity in many bacteria. The main component of the flagellar filament, flagellin (FliC), often undergoes post-translational modifications, with glycosylation being a common occurrence. In *Pseudomonas aeruginosa* PAO1, the b-type flagellin is *O*-glycosylated with a structure that includes a deoxyhexose, a phospho-group, and a previous unknown moiety. This structure resembles the well-characterized glycan (Type A) in *Clostridioides difficile* strain 630, which features an *N*-acetylglucosamine linked to an *N*-methylthreonine via a phosphodiester bond. This study aimed to characterize the b-type glycan structure in *Pseudomonas aeruginosa* PAO1 using a set of mass spectrometry experiments. For this purpose, we used wild-type *P. aeruginosa* PAO1 and several gene mutants from the b-type glycan biosynthetic cluster. Moreover, we compared the mass spectrometry characteristics of the b-type glycan with those of *in vitro* modified Type A-peptides from *C. difficile* strain 630Δ*erm*. Our results demonstrate that the thus far unknown moiety of the b-type glycan in *P. aeruginosa* consists of an *N,N*-dimethylthreonine. These data allowed us to refine our model of the flagellin glycan biosynthetic pathway in both *P. aeruginosa* PAO1 and *C. difficile* strain 630.

Bacterial flagella are intricate, whip-like appendages that extend from the cell bodies of many motile bacteria, playing a crucial role in their locomotion and environmental navigation.^1^ These structures are not only essential for bacterial motility but also contribute significantly to pathogenicity, colonization, and biofilm formation.^2,3^ At the core of the flagellar filament is flagellin, also known as FliC, a highly conserved protein across many bacterial species that polymerizes to form the helical structure driving bacterial movement.^4^

Beyond its structural role, flagellin undergoes various post-translational modifications,^5^ among which glycosylation is particularly important.^6^ Glycosylation of flagellin can affect the assembly and function of flagella, influencing the stability, flexibility, and overall performance of the flagellar filament. This modification is not uniform across bacterial species; different bacteria employ distinct glycosylation patterns, which can affect their motility in various ways.^7,8^ Moreover, flagellin glycan structures can vary between different strains of the same species.

Flagellin glycosylation is also observed in *P. aeruginosa*, a facultative anaerobic, Gram-negative bacterium that primarily causes infections in immunocompromised individuals or patients receiving intensive care.^9,10^ Importantly, *P. aeruginosa* strains without flagellin glycosylation showed attenuated virulence.^11^ In the *P. aeruginosa* PAK strain, the a-type flagellin is *O*-glycosylated with a structure comprising 11 monosaccharide residues, including a deoxyhexose at the base.^12,13^ In contrast, the b-type flagellin, as observed in the *P. aeruginosa* PAO1 strain, is decorated with a very different structure Figure 1A that consists of a single *O*-linked deoxyhexose, likely a l-rhamnose,^14^ linked to an unknown moiety through a phosphodiester bond.^15^ Initial experiments indicated that the unknown moiety consists of a tyrosine,^16^ however subsequent studies by mass spectrometry^15^ determined a mass for this moiety (129 Da) which is not compatible with a tyrosine. Hence, the nature of the unknown moiety has been a longstanding unsolved question.

**Figure 1:**
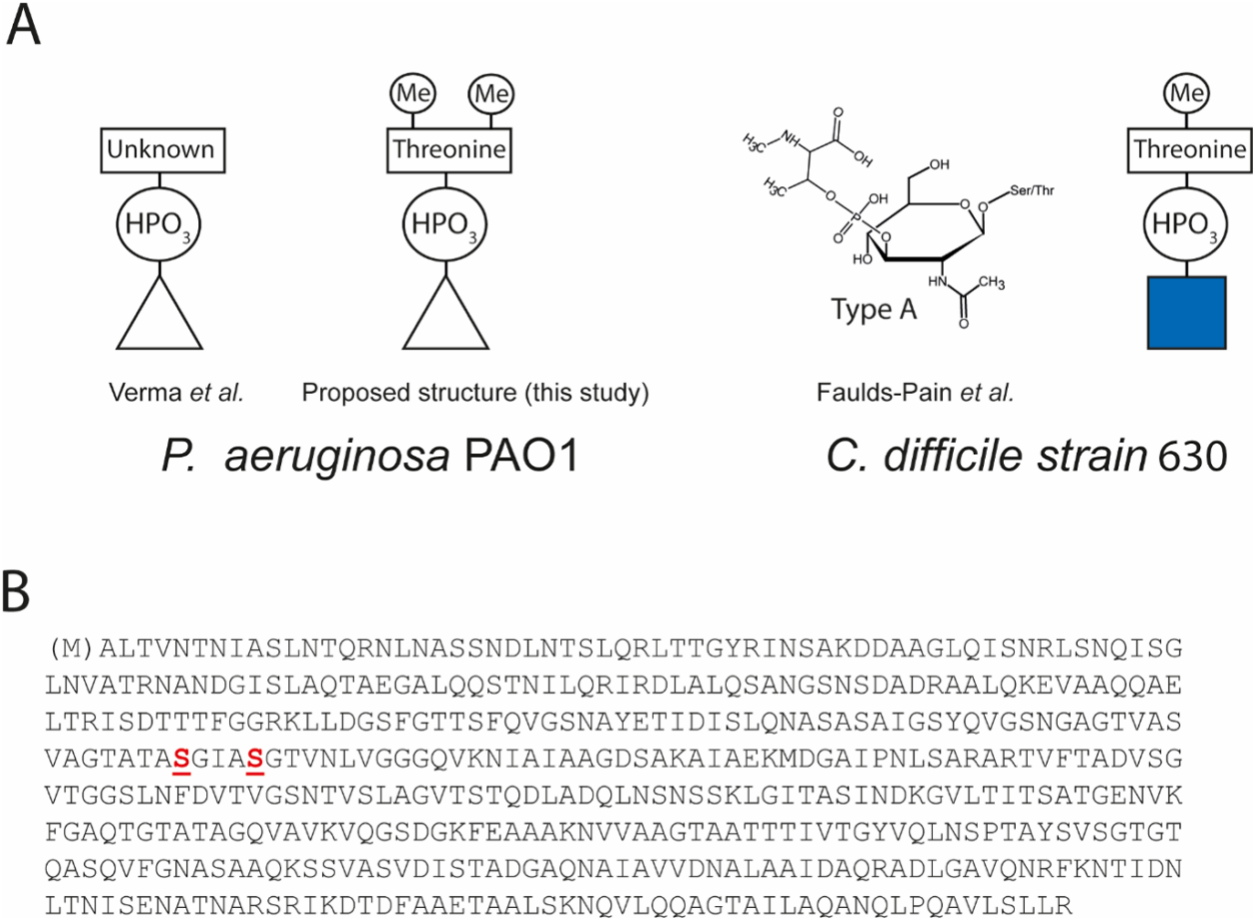
Flagellin glycan structures in *Pseudomonas aeruginosa* PAO1 and *Clostridioides difficile* strain 630. (A) Schematic structure of the described^15^ and proposed structure of the b-type glycan in *P. aeruginosa* and the molecular and schematic structure of the Type A glycan in *C. difficile* strain 630^17^ Triangle: deoxyhexose (most probably a rhamnose (based on (14))). Blue square: *N*-acetylglucosamine. HPO_3_: phospho. Me: methyl. (B) Primary sequence of b-type flagellin of *P. aeruginosa* PAO1 (flagellin, UniprotID: P72151). The glycosylated serine residues as determined by Verma et al.^15^ are highlighted in red, and underlined. The N-terminal methionine is lacking in the mature protein.

Interestingly, the glycan structure in *P. aeruginosa* has overlapping features to a glycan structure which is found on flagellin in several *Clostridioides difficile* strains. This glycan structure (Type A, Figure 1A) consists of an *O*-linked *N*-acetylglucosamine (GlcNAc), that is linked to *N*-methyl-l-threonine through a phosphodiester bond.^17,18^ In support of the structural similarity between the two species, gene clusters encoding enzymes with similar predicted activities are found in both genomes (Figure S1).^15,17,19^ The incomplete characterization of the b-type glycan in *P. aeruginosa* is a significant gap in knowledge and a crucial step toward fully understanding the similarities between the two species.

Recently, we have observed that a small fraction of the Type A structure in *C. difficile* is modified, and one of the observed alterations was predicted to be a structure comprising an extra methyl group on the threonine i.e., *N,N*-dimethylthreonine.^20^ Such a structure would be compatible with what is currently known about the terminal part of the b-type glycan on *P. aeruginosa* flagellin, i.e., a mass of 129 Da. Hence, we hypothesized that the unknown moiety of the b-type glycan on *P. aeruginosa* flagellin is a *N,N*-dimethylthreonine^20^ (Figure 1A). Here, using mass spectrometry-based analyses, we provide strong evidence that this structure is indeed part of the glycan structure on *P. aeruginosa* PAO1 flagellin.

## Results

### Mass Spectrometric Analysis of b-type Glycan-Modified Flagellin of *P. aeruginosa* PAO1 is Consistent with the Presence of a *N*,*N* Dimethylthreonine as Part of the Glycan Structure

**Figure 2:**
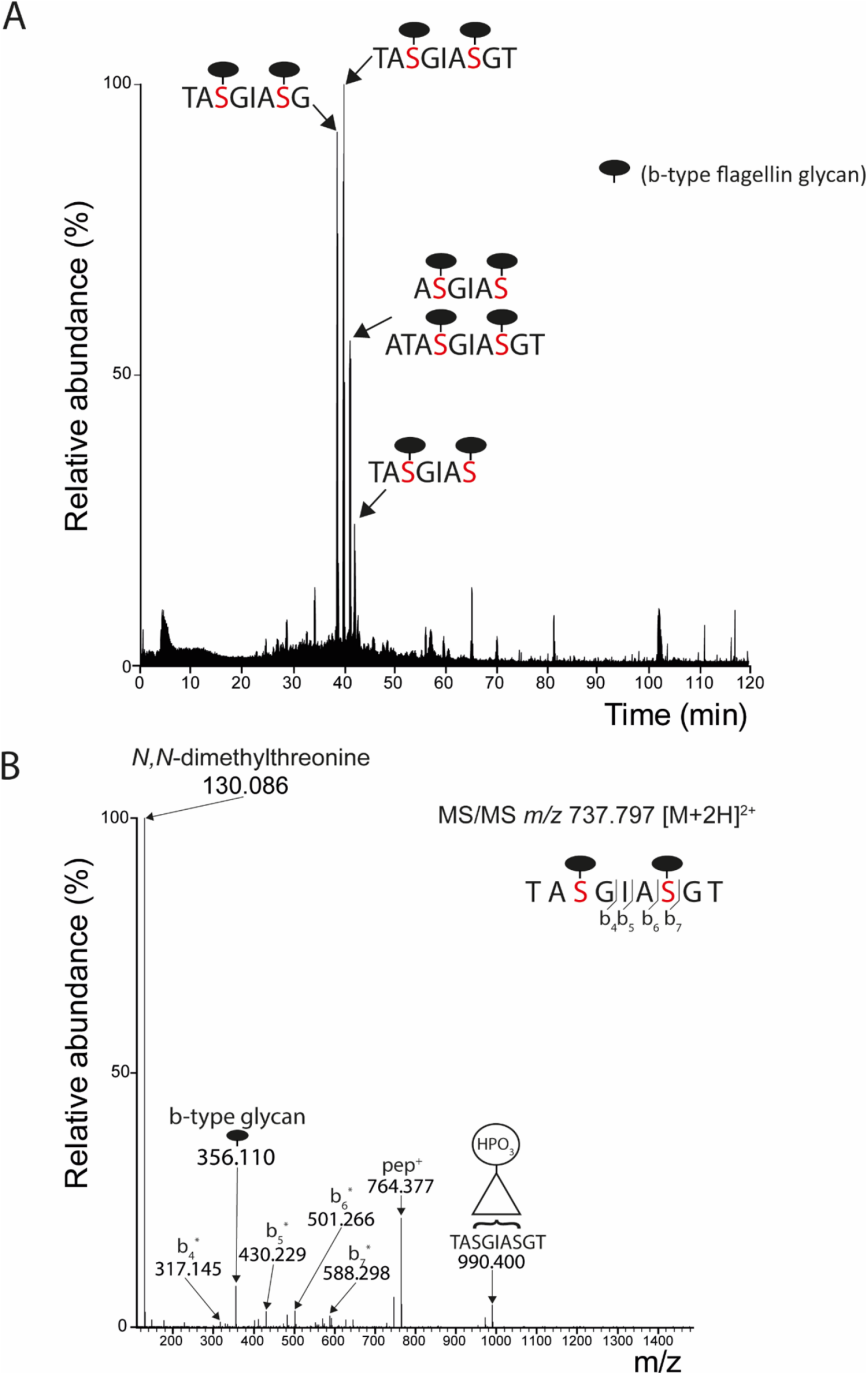
Mass spectrometry analysis of b-type glycan-modified flagellin peptides from *P. aeruginosa* PAO1 after IMAC purification. (A) Total ion chromatogram of an LC-MS/MS analysis of IMAC-purified Proteinase K peptides from *P. aeruginosa* PAO1. The major b-type glycan-modified peptides corresponding to the observed peaks are indicated. See also Table 1. (B) MS/MS spectrum of the Proteinase K flagellin peptide TASGIASGT, modified with two type-b glycans. All observed b-ions have lost the b-type glycan (indicated with a *).

A previous study showed that b-type flagellin from *P. aeruginosa* PAO1 is glycosylated at Ser-191 and Ser-195 in the mature protein,^15^ (Figure 1B). For these experiments, purified flagella were used. To study the *P. aeruginosa* PAO1 flagellin glycosylation, we sought to apply an approach i.e., Immobilized Metal Affinity Chromatography (IMAC), that is used to affinity purify phosphorylated peptides in phosphoproteomics studies. Recently, we found that this method can also be used to enrich for Type A-modified tryptic flagellin peptides from *C. difficile*(20) and we believed that this is due to the presence of the phospho-moiety in the Type A structure (Figure 1B). Because this is also present in the type b-glycan of *P. aeruginosa* PAO1 (Figure 1A), we predicted that this approach would also enrich b-type glycan-modified peptides. A Proteinase K digest of the *P. aeruginosa* PAO1 proteome was performed to generate small peptides that would allow us to pinpoint the modified residues. Following IMAC purification, peptides were analyzed by liquid chromatography and tandem mass spectrometry (LC-MS/MS). The most intense peaks in the LC-MS/MS data represented various b-type glycan-modified peptides originating from flagellin (Figure 2A).

The smallest peptide (ASGIAS) allowed us to infer that Ser-191 and Ser-195 of flagellin are likely modified with the b-type glycan as previously reported by Verma et al.^15^ Based on the mass of the five major glycopeptides (Figure 2A), a neutral mass of 355.104 Da for the b-type glycan was observed (Table 1).

**Table 1:** Probing the b-type Glycan Based on the MS Analysis of Proteinase K Generated b-type Glycan-Modified Flagellin Peptides from *P. aeruginosa*.^a^

observed *m*/*z*	charge	observed mass (Da)	peptide	peptide mass (Da)	mass b-type glycan (Da)
687.273	2+	1373.539	TA**S**GIA**S**G	663.331	355.104
737.797	2+	1474.587	TA**S**GIA**S**GT	764.379	355.104
773.315	2+	1545.623	ATA**S**GIA**S**GT	835.416	355.104
608.239	2+	1215.471	A**S**GIA**S**	505.262	355.105
658.762	2+	1316.517	TA**S**GIA**S**	606.309	355.104

a On each peptide, two b-type glycans were found (serines indicated in bold and underlined). See also Figure 2.

These results are consistent with our hypothesis that the unknown moiety is a *N,N*-dimethylthreonine (theoretical neutral mass of predicted b-type glycan is 355.103 Da (Figure 1A)). MS/MS analysis of the peptide TASGIASGT with two b-type glycans (Figure 2B) showed the loss of the full type-b glycan at *m*/*z* 356.110 ([M + H^+^]^+^). The MS/MS data also showed a prominent ion at *m*/*z* 130.086 ([M + H^+^]^+^) (Figure 2B). In line with the proposed b-type glycan structure (Figure 1A), and our previous work,^20^ this ion was tentatively assigned as *N,N*-dimethylthreonine. Of note, MS3 experiments showed that the ion at *m*/*z* 130.086 was derived from the b-type glycan (Figure S2).

### *P. aeruginosa* PAO1 *pa1088*-*pa1091* Mutant Strains Exhibit Truncated b-type Glycan Structures

In *C. difficile* strain 630, the biosynthesis of the Type A glycan depends on *cd0240* (encoding a glycosyltransferase) and the adjacent *cd0241-cd0244* operon.^17^ In the *P. aeruginosa* PAO1 biosynthetic gene cluster (Figure S1), only four genes are found (*pa1088*-*pa1091*) because the glycosyltransferase and *cd0244* homolog are organized in a single gene (*pa1091*). To study the role of the individual genes, we recently analyzed the Type A glycan structure and variations thereof in *C. difficile* strain 630 *cd0241-cd0244* mutant strains using a quantitative proteomics experiment.^19^ In the *cd0241, cd0242*, and *cd0244* mutant strains, truncated Type A structures consisting of only the core GlcNAc were observed. In the *cd0243* mutant, Type A structures solely lacking the methyl group were found, which is in line with the putative methyltransferase activity of CD0243. The methyltransferase homolog in *P. aeruginosa* PAO1 is *pa1088* (Figure S1). Hence, based on our proposed b-type glycan structure (Figure 1A), we predicted a truncated b-type glycan lacking two methyl groups in a *pa1088* mutant strain. To test this hypothesis, we performed a quantitative proteomics experiment using *P. aeruginosa* PAO1 wildtype (WT) and *pa1088-pa1091* mutant strains. To increase the homogeneity in peptides, we used a combination of trypsin and chymotrypsin instead of Proteinase K for these experiments. All strains were analyzed in triplicate.

First, the sample was affinity purified using IMAC. The digestion protocol resulted in the tryptic+chymotryptic peptide QVGSNGAGTVASVAGTATASGIASGTVNLVGGGQVK (aa 172-207) carrying two b-type glycans.

**Figure 3:**
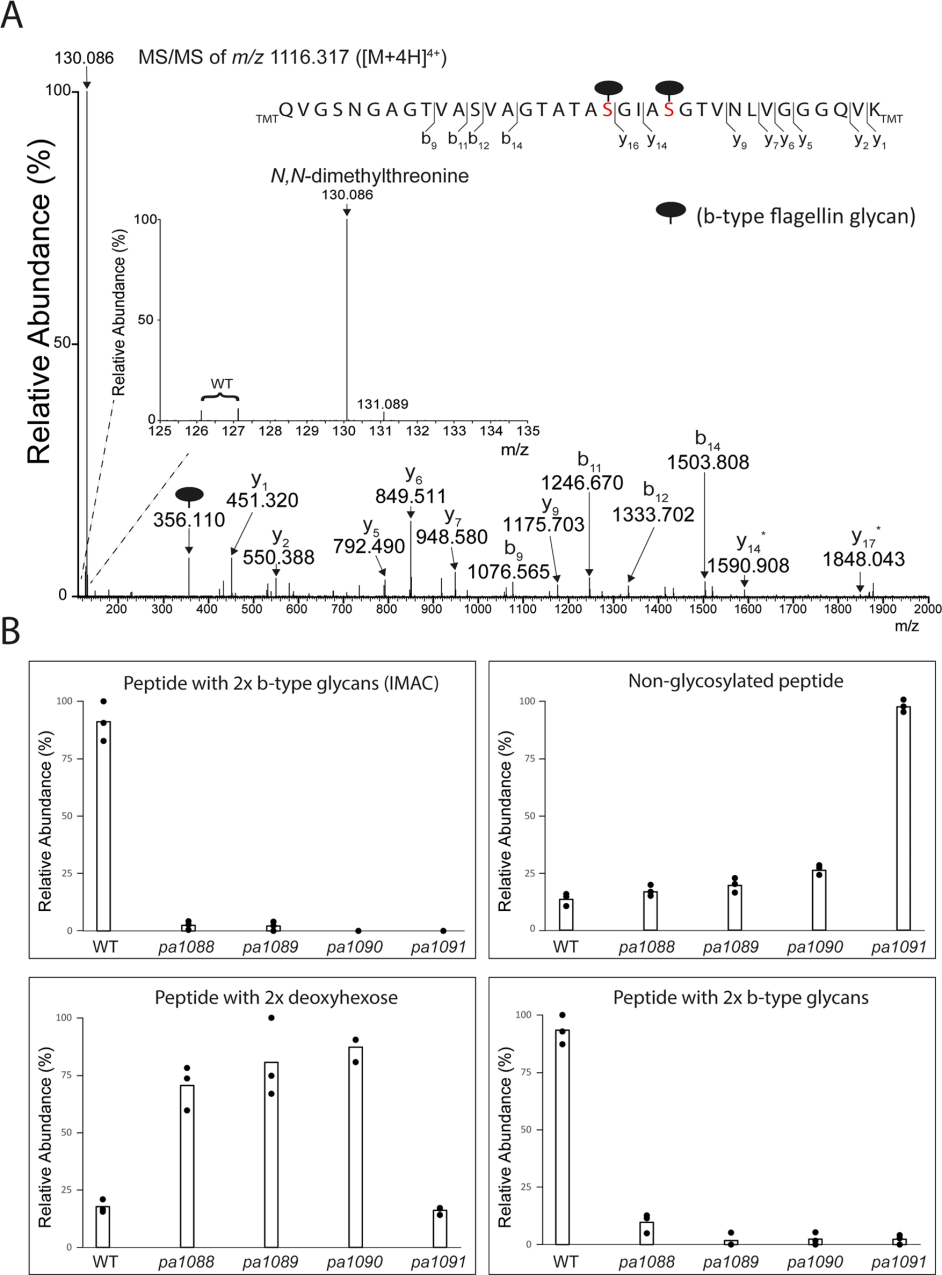
Analysis of variations of the b-type glycan structure in *P. aeruginosa* PAO1 WT and mutant strains. (A) Protein extracts of WT and pa1088-pa1091 *P. aeruginosa* PAO1 (mutant) strains were digested with a combination of trypsin and chymotrypsin. All strains were analyzed in triplicate. Following TMT-labeling, the sample was affinity purified using IMAC and analyzed by LC-MS/MS. Shown is the MS/MS spectrum of the peptide QVGSNGAGTVASVAGTATASGIASGTVNLVGGGQVK with two b-type glycan structures. The inset depicts the zoom-in of the TMT-reporter region, which also shows the b-type glycan-specific fragment at *m*/*z* 130.086. Fragment ions indicated with an * have lost the b-type glycans. (B) Quantification of the different variants of the QVGSNGAGTVASVAGTATASGIASGTVNLVGGGQVK flagellin peptide in WT and individual *P. aeruginosa* PAO 1 mutant strains, i.e., carrying two b-type glycans (top left panel (IMAC purified) and lower right panel (without IMAC), nonglycosylated (top right panel) and carrying two deoxyhexoses (lower left panel)). Quantification was based on the relative intensity of the corresponding TMT-reporter ions. Dots indicate the relative intensity for each biological replicate.

This peptide was observed at *m*/*z* 1116.317 ([M + 4H^+^]^4+^. Again, the MS/MS spectrum Figure 3A) showed the b-type glycan-specific ions at *m*/*z* 130.086 and 356.110. Zooming in on the TMT-reporter region (Figure 3A (inset) and Figure 3B (upper left panel)) showed that this peptide was observed in the WT samples (TMT-reporter 126, 127N, and 127C) but not in the mutant strains. Next, we looked for the same peptide with b-type glycans lacking two methyl groups, which we would expect in the *pa1088* mutant strain, but such a peptide was not observed. Also, peptides with b-type glycans lacking one methyl group were not identified. As expected, given the IMAC affinity purification, mutants with truncated structures lacking the phospho-moiety, e.g., nonglycosylated or only consisting of the deoxyhexoses, were not observed. To test whether such peptides were present in the *pa1088* mutant and the other strains, we also analyzed the sample without the IMAC affinity purification step. For this purpose, the full TMT-labeled digest was fractionated in 12 fractions using high-pH reversed phase chromatography, and each fraction was analyzed by LC-MS/MS.

First, we explored our proteomics data for PA1088-PA1091 to see if we could confirm the mutant phenotype and to investigate if there were strong polar effects, i.e., expression effects on genes downstream of the target gene,^21^ as we previously observed with *C. difficile* insertional mutants.^19^ For PA1088-PA1090, only a few (quantifiable) peptides were found and the quantitative information based on the relative intensity of the reporter ions was not consistent with the mutant phenotype, even though the data indicated lower levels of PA1088 and PA1090 in the corresponding mutant strains (Figure S3). We observed a higher protein coverage for PA1091 but the quantitative proteomics data could not confirm the mutant phenotype (Figure S3). It is known from insertional mutants that the region upstream of the insertion can still be translated. Therefore, we mapped the identified peptides from PA1091 on the full sequence (Figure S4) and checked the relative levels of each peptide as compared to the control. This showed that peptides covering the C-terminal region (downstream of the transposon insertion) were indeed found at lower levels, while peptides covering the N-terminal region were not, indicating that also in the *pa1091* mutant, part of the open reading frame is translated, but this does not result in an active protein. Of note, the peptides that were used for quantification of PA1088-PA1089 were all downstream from the transposon insertion in the corresponding mutant strain. Compared to our previous experiments with *C. difficile*,^19^ no strong polar effects were apparent (Figure S3), although based on a single peptide some polar effects may be present in the *pa1089* mutant strain.

Next, we checked the data for the different variations in the b-type glycan on the tryptic+chymotryptic peptide QVGSNGAGTVASVAGTATASGIASGTVNLVGGGQVK, i.e., the nonglycosylated peptide and the peptide with truncated b-type glycan consisting of only the core deoxyhexoses. Both were identified with MS/MS spectra that look very different from the b-type glycan-modified peptides because they lacked the prominent b-type glycan-specific ions at *m*/*z* 130.086 and 356.110 (Figure S5). On the other hand, the peptide-specific fragments largely overlapped. Based on the TMT-reporter intensities in these spectra, the nonglycosylated peptide was predominantly observed in the *pa1091* mutant strain (Figure 3B, upper right panel). In contrast, the peptide with a truncated b-type glycan consisting of only the core deoxyhexoses was enriched in the *pa1088*, *pa1089*, and *pa1090* mutant strains (Figure 3B, lower left panel). The fully glycosylated peptide was also identified in the overall proteomics data set and the quantification data (Figure 3B, lower right panel) agreed with the data from the IMAC purified material (Figure 3B, upper left panel). The peptide with b-type glycans lacking one or two methyl groups was also not observed in the overall proteomics data.

Collectively, our data show that insertional mutagenesis of genes *pa1088*-*pa1091* in *P. aeruginosa* PAO1 leads to aberrant glycosylation of b-type flagellin. In three mutants, i.e., *pa1088*-*pa1090*, only deoxyhexoses were observed, while nonglycosylated flagellin was found in the *pa1091* mutant strain.

### Tandem Mass Spectrometry-Based Assignment of *N,N*-Dimethylthreonine as Part of the b-type Glycan in *P. aeruginosa*

Because the *pa1088* mutant data shown above did not demonstrate b-type glycan structures lacking methyl groups, we sought an alternative approach to substantiate our assignment of the *N,N*-dimethylthreonine as part of the b-type glycan. We reasoned that *in vitro* methylation of the Type A structure in *C. difficile* should generate a glycan structure identical to our proposed b-type glycan in *P. aeruginosa*, except for the monosaccharide (Figure 1A). We hypothesized that the mass spectrometric fragmentation characteristics of both structures should be highly similar.

To test this, we methylated tryptic peptides from a WT *C. difficile* 630Δ*erm* strain by reductive amination. With this protocol, all peptide N-termini and lysine side chains were dimethylated. The Type A glycan already contains an *N*-methylated threonine (Figure 1A); therefore, only one methyl group was added resulting in a modified Type A structure with two methyl groups.

**Figure 4:**
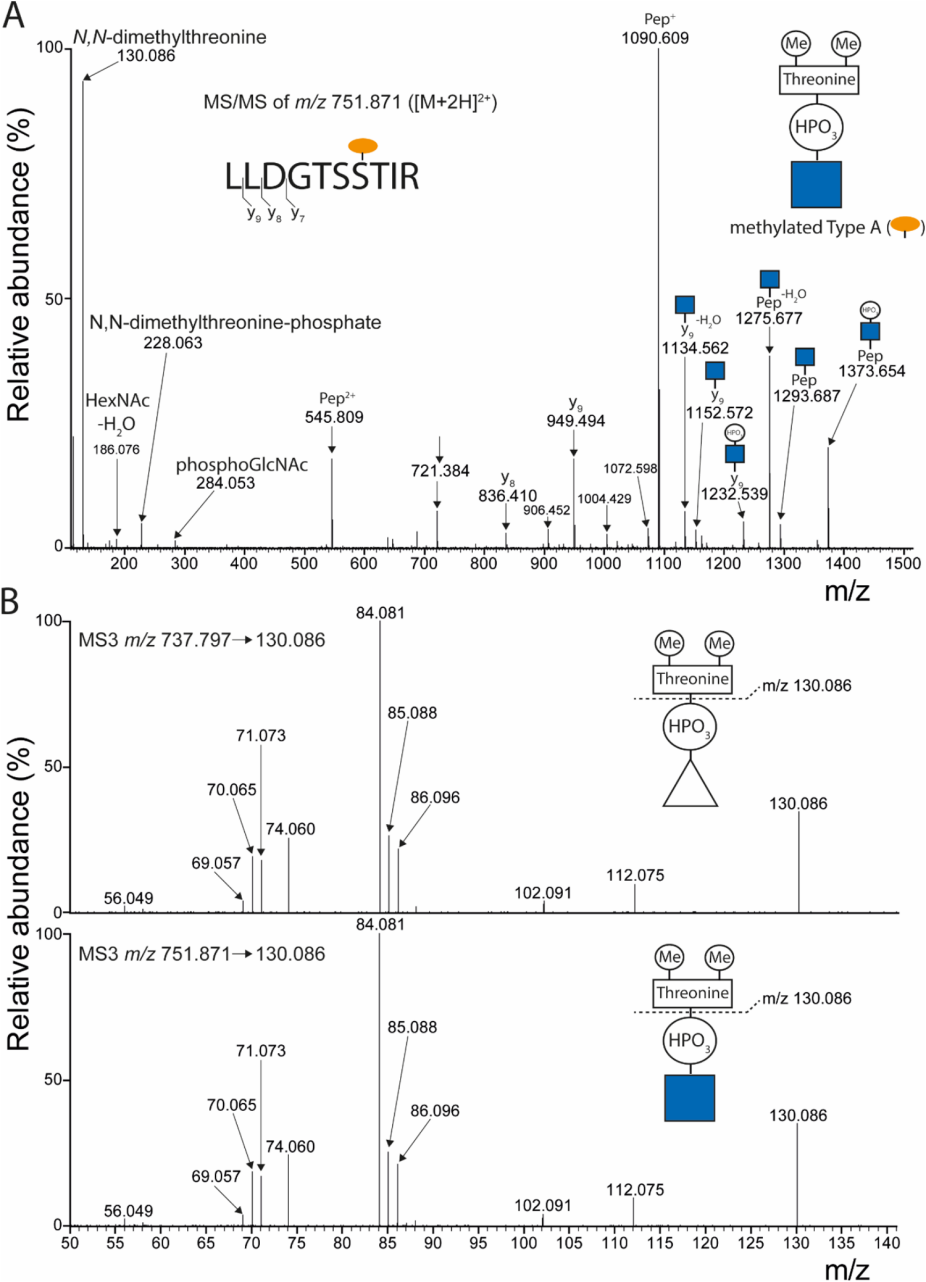
Mass spectrometric identification of *N,N*-dimethylthreonine as part of the b-type glycan in *P. aeruginosa* PAO1. (A) A tryptic digest of *C. difficile* strain 630Δ*erm* proteins was *in vitro* methylated using reductive amination and analyzed by LC-MS/MS. Shown is the MS/MS spectrum of the methylated Type A-modified tryptic flagellin peptide LLDGTSSTIR (site assignment based on (17)). The fragment ion at *m*/*z* 130.086 corresponds to *N*,*N*-dimethylthreonine. (B) Comparison of the fragmentation pattern of the ion at *m*/*z* 130.086 generated by fragmentation of a *P. aeruginosa* type-b modified peptide and a methylated Type A-modified *C. difficile* peptide, respectively. Upper panel: Fragmentation (MS3) of the ion at *m*/*z* 130.086 generated by MS/MS of the b-type glycan modified peptide TASGIASGT from *P. aeruginosa* flagellin (see Figure 2B). Lower panel: Fragmentation (MS3) of the ion at *m*/*z* 130.086 generated by MS/MS of the methylated Type A-modified peptide LLDGTSSTIR from (C) difficile flagellin (see panel A).

During the LC-MS/MS analysis, we focused on one of the tryptic flagellin peptides from *C. difficile* (LLDGTSSTIR) with the methylated Type A structure (Figure 4A). Besides the peptide-specific fragments, a few differences with a b-type glycan-modified peptide from *P. aeruginosa* were apparent in the MS/MS data. First of all, the loss of the fully methylated Type A moiety, which would result in a fragment at *m*/*z* 413.132 ([M + H^+^]^+^) was hardly observed (Figure 4A). Instead, partial fragmentation of the methylated Type A was apparent. For example, a fragment at *m*/*z* 228.063 ([M + H^+^]^+^) corresponding to an *N,N*-dimethylthreonine-phosphate was observed. The corresponding fragment at *m*/*z* 214.048, lacking one methyl group, is well-known from the fragmentation spectra of WT Type A-modified peptides.^18,20^ Moreover, partial fragmentation of the methylated Type A was observed by fragments at e.g., *m*/*z* 1275.677, 1293.687, and 1373.654.

Most importantly, the MS/MS spectrum showed the prominent ion at *m*/*z* 130.086 ([M + H^+^]^+^). In this case, it could be confidently assigned as corresponding to *N,N*-dimethylthreonine.

Finally, we performed MS3 experiments to investigate whether the fragmentation patterns of the ion at *m*/*z* 130.086 ([M + H^+^]^+^) generated from a WT *P. aeruginosa* b-type glycan-modified peptide (Figure 2B) and the methylated Type A-modified peptide from *C. difficile* (Figure 4A) are the same. As shown in Figure 4B, these spectra are identical, providing strong evidence that the hitherto unknown moiety in the *P. aeruginosa* b-type glycan is an *N,N*-dimethylthreonine.

## Discussion

The results from our study strengthen the notion that the b-type glycan in *P. aeruginosa* PAO1 and the Type A glycan in *C. difficile* strain 630 are very similar. Besides the difference in the core monosaccharide, the only difference in composition is the degree of *N*-methylation of the threonine as part of the glycan structure, i.e., in the b-type glycan in *P. aeruginosa* PAO1 it is dimethylated, while in the Type A glycan in *C. difficile* it is monomethylated.

The *m*/*z* of the *P. aeruginosa* b-type glycan-specific ion at *m*/*z* 356.110 that we observed in the MS/MS data is in agreement with previous data.^15^ Also in the earlier study, a fragment ion at *m*/*z* 130.1 on a low mass resolution instrument was observed in the MS/MS spectra of type-b glycan-modified peptides, but the identity was not elucidated.^15^ For our assignment, the data on the fragmentation of this ion, and comparison with methylated Type A from *C. difficile* was pivotal. Interestingly, several major fragments that we observed in the MS3 spectra, e.g., at *m*/*z* 70, 74, 84, 85, 86, 102, and 112, were observed in an MS/MS spectrum of *N,N*-dimethylthreonine analyzed after CID fragmentation on a Q-TOF instrument.^22^ In the Type A glycan in *C. difficile*, the *N*-methylthreonine-phospho moiety is linked to the O-3 position on the GlcNAc. NMR analysis is necessary to determine whether the *N*,*N*-methylthreonine-phospho moiety in the *P. aeruginosa* b-type glycan is also in the same position on the deoxyhexose.^14^

Our results confirm the b-type glycan site assignment on flagellin. Proteinase K digestion resulted in a set of b-type glycan-modified peptides with two b-type glycans and one of these (ASGIAS) had only two possible *O*-glycosylation sites, corresponding to Ser-191 and Ser-195 in the mature protein. Previously, these sites were determined based on the mass spectrometry analysis of a b-type glycan-modified peptide after β-elimination.^15^

**Figure 5:**
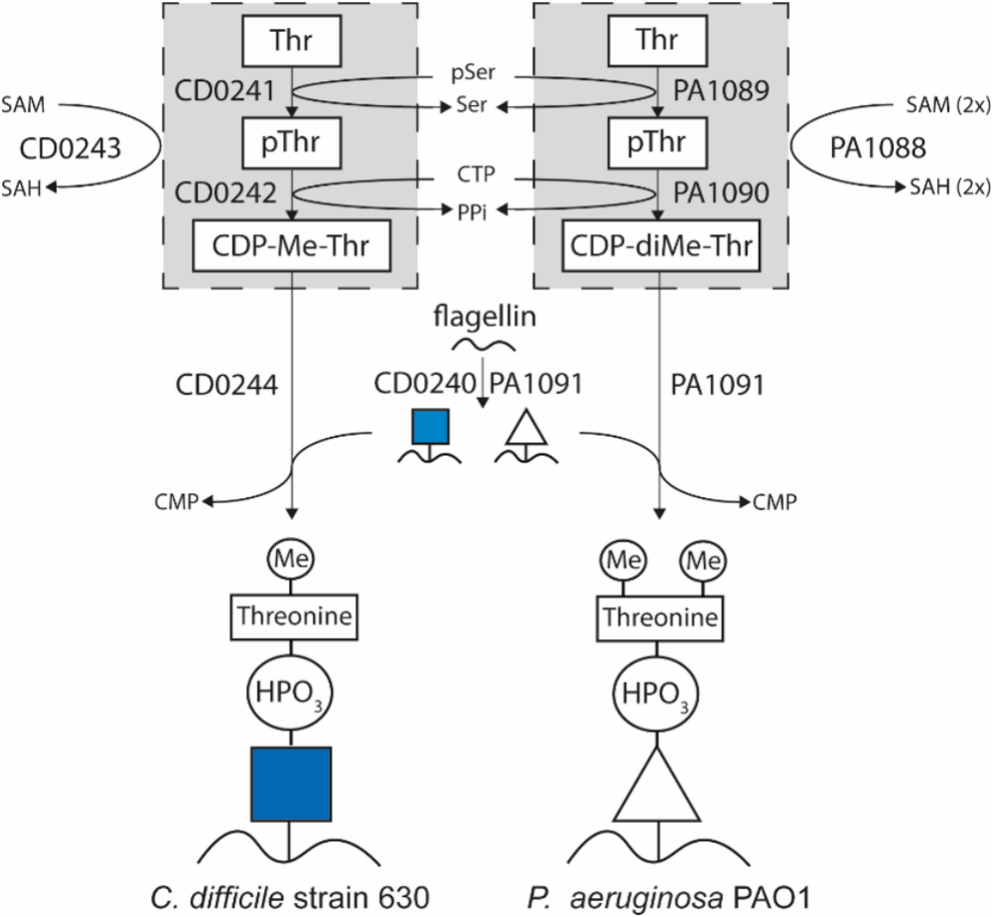
Schematic representation of the model for the biosynthesis of the flagellar glycans in *C. difficile* strain 630 and *P. aeruginosa* PAO1. For details about the individual steps and bioinformatic predictions of enzyme activities, see Claushuis et al.^19^ pThr/pSer: phosphothreonine/serine. Me: *N*-methyl, diMe: *N*,*N*-dimethyl. CTP/CDP/CMP: Cytidine 5′-tri/di/monophosphate. PPi: pyrophosphate. SAM: *S*-adenosylmethionine. SAH: *S*-adenosylhomocysteine. Blue square: *N*-acetylglucosamine. Triangle: deoxyhexose.

Recently, we presented a revised model for the Type A biosynthesis in *C. difficile* strain 630^19^ based on a mass spectrometry-based proteomics experiment with mutant strains and bioinformatic analyses. The different steps in this model are schematically presented in Figure 5.

In this model, we predicted a novel biosynthetic intermediate, CDP-threonine,^19^ whose synthesis and subsequent role as a donor substrate is supposed to be controlled by CD0242 and CD0244, respectively. In *C. difficile*, the loss of CD0243 activity (a putative methyltransferase) partially resulted in a structure lacking the methyl group but our experimental approach did not allow us to determine the timing of the methylation event. The truncated b-type glycan in the putative methyltransferase (*pa1088*) *P. aeruginosa* mutant strain only consisted of the core deoxyhexose, in line with previous observations.^15^ Structures lacking methyl groups were not observed in this mutant strain, suggesting that methylation is a crucial, and early event in the b-type glycan biosynthetic pathway. Based on this finding, we offer the testable hypothesis that methylated intermediates are the preferred substrates for one or more of the enzymes in the biosynthesis routes. Hence, we here refine our model and postulate that CDP-*N*-methylthreonine and CDP-*N,N*-dimethylthreonine are the *in vivo* donor substrates of the reaction catalyzed by CD0244 and PA1091, respectively (Figure 5). The difference in the degree of methylation could be related to the activity of the methyltransferases involved i.e., PA1088 and CD0243. It is known that some *N*-methyltransferase reactions result in monomethylation, while others in di- or even trimethylation.^23,24^ The fact that a small fraction of the Type A glycan in *C. difficile* also has an extra methyl group,^20^ suggests that CD0243 activity can also proceed toward dimethylation. Of note, in addition to the structure lacking the methyl group, structures comprising only the core GlcNAc were also enriched in the *cd0243* mutant strain in *C. difficile*.^19^ We argued that this was due to polar effects in this strain but, assuming our model is correct, it might well be that the reaction with CDP-threonine as a donor substrate is suboptimal.

Because PA1091 encodes for both glycosyltransferase (FgtA, belonging to the GT2 family) as well as phosphotransferase activity, it is unfortunately difficult to dissect the role of both activities independently, although a point mutation in the catalytic side of the phosphotransferase would be an interesting option. PA1090, a homolog of CD0242, is predicted to be responsible for the biosynthesis of CDP-(*N,N*-dimethyl)threonine. In line with this, we observed that the truncated b-type glycan in the corresponding mutant only contained the core monosaccharide. As expected, we also observed this phenotype in the *pa1089* mutant. Interestingly, previous data showed a mixture of WT structures and structures containing only the deoxyhexoses in a *pa1089* mutant.^15^ We have no explanation for this apparent discrepancy with our data other than that the mutant was generated using a different method.

In our quantitative proteomics data, we only observed a limited number of peptides corresponding to the enzymes involved in the b-type glycan biosynthesis, hampering reliable quantification. In *C. difficile* these proteins were readily identified and quantified,^19^ indicating that their overall cellular levels are higher. This might be related to the fact that *C. difficile* has multiple flagella per cell and more sites in flagellin that are modified with the Type A glycan, whereas *P. aeruginosa* has only one flagellum per cell and only two modifications per flagellin molecule. Ratio compression, known from TMT-based quantification methods, could also have played a role in our quantitative analyses. This is probably why the absence of the type-B modified peptide in the mutant strains was more apparent following IMAC purification. The minor residual signals that were observed in the *pa1088* and *pa1089* mutant strains in the IMAC data can largely be explained by impurities in the TMTpro labels; i.e., each label contains a small percentage of different isotopologues (TMT Reporter Ion Isotope Distributions for TMTpro 16plex batch WK334339, Thermo Fisher Scientific). The fact that we could use IMAC for affinity purification of b-type glycan-modified peptides demonstrated that the recently presented method^20^ is more broadly applicable.

Both the innate and the adaptive immune response to flagellin is well-documented.^25,26^ Interestingly, a *P. aeruginosa* flagella vaccine showed promising results in cystic fibrosis patients, highlighting the flagellin protein as a potential vaccine candidate.^27^ Our data may contribute to future vaccine designs, even though recent data suggested that the b-type glycan is less important than the a-type glycan for the induction of protective antibodies.^28^ Moreover, given the importance of the *P. aeruginosa* PAO1 flagellin glycosylation for virulence,^11^ further understanding and characterization of the enzymes involved in the b-type glycan biosynthesis may lead to the development of potential inhibitors, some of which may even be able to target *P. aeruginosa* as well as *C. difficile* strains.

## Conclusions

Bacterial motility largely relies on cell surface flagella, complex locomotive structures composed of various protein subunits. The flagellar filament is primarily made up of polymeric flagellin (flagellin). In many bacteria, flagellin undergoes post-translational modifications, often glycosylation, which can vary significantly between and within species. In the pathogenic bacterium *P. aeruginosa*, flagellin glycosylation plays a crucial role in virulence. Nonetheless, the complete composition of the b-type flagellin glycan in *P. aeruginosa* PAO1 has long been an open question. Here, through a series of mass spectrometry experiments, we identify the previously uncharacterized component of the b-type glycan as *N,N*-dimethylthreonine, which is linked to deoxyhexose via a phosphodiester bond. Our findings further emphasize the similarities between the b-type flagellin glycan in *P. aeruginosa* and the Type A glycan observed in *C. difficile*. Based on these results, we present for the first time a testable model for the biosynthesis of the b-type glycan in *P. aeruginosa* PAO1.

## Methods

### Bacterial Strains and Culturing Conditions

The wild-type *P. aeruginosa* PAO1 strain was a generous gift from prof. A. Briegel from the Institute of Biology Leiden (IBL) in Leiden, The Netherlands. The PAO1 mutant strains were ordered from the Salipante Lab at the University of Washington, USA.^29,30^ The transposon insertion site of all mutants had been confirmed via Sanger sequencing of individual colonies. Information about the mutant strains can be found in Table S1. Cells were plated on Luria–Bertani (LB) agar plates. Three colonies per plate (representing three biological replicates per strain) were inoculated in 5 mL LB medium and grown for 16 h at 37 °C, while rotating at 180 rpm. *C. difficile* strain 630Δ*erm* was cultured as described previously.^19^

### Protein Extraction, Reduction Alkylation, Digestion and TMTpro-Labeling

Following cell culturing, cells were pelleted by centrifugation (3220*g*, 10 min, 4 °C), resuspended in 5 mL of ice-cold phosphate-buffered saline (PBS), and centrifuged again. The washing step was repeated once more. After the last wash, pellets were resuspended in 1 mL of ST lysis buffer (5% SDS, 0.1 M Tris-HCl pH 7.5) and incubated on ice for 20 min. Then, cells were lysed using sonication for 30 s, followed by 30 s of cooling on ice. This was repeated five times. After sonication, samples were centrifuged at 10,000*g* for 15 min at room temperature (RT), and the supernatant was collected. Protein levels were quantified using a bicinchoninic acid (BCA) assay (Pierce Rapid Gold BCA Protein Assay). For reduction and alkylation of cysteines, 1 μL 0.5 M tris(2-carboxyethyl)phosphine (final concentration 5 mM), 3 μL 0.3 M iodoacetamide (final concentration 9 mM), and 2 μL 0.5 M dithiothreitol (final concentration 10 mM) were sequentially added to a protein solution (100 μg in 100 μL ST buffer), with each step incubated for 30 min at RT. Then proteins were precipitated by sequentially adding 400 μL methanol, 100 μL chloroform, and 300 μL water, with vortexing after each step. Following centrifugation at max speed in an Eppendorf 5424 R centrifuge, the protein pellet at the interface was collected and washed three times with 500 μL methanol. The pellet was then air-dried at 37 °C.

For Proteinase K (Merck) digestion, a protein pellet corresponding to 100 μg protein was reconstituted in 100 μL 100 mM ammonia solution containing 4 μg Proteinase K, after which samples were incubated overnight at 37 °C. For the combined trypsin (Worthington Biochemical)/chymotrypsin (Worthington Biochemical) digestion, a protein pellet corresponding to 100 μg protein was resuspended in 100 μL 40 mM HEPES pH 8.0 containing 2 μg trypsin and 2 μg chymotrypsin, and incubated overnight at 37 °C, after which an additional 2 μg of each enzyme was added. Samples were incubated for two hours. Tandem Mass Tagging (TMTpro, Thermo) labeling was then performed using 10 μg of tryptic+chymotryptic peptides from each strain. For the overview of the labels for each strain, see Table S2. Following mixing of the samples and freeze-drying, peptide fractionation was performed as described previously.^19^ Briefly, peptides were resuspended in 10 mM ammoniumbicarbonate pH 8.4 (mobile phase A) and separated on an Agilent Eclipse Plus C18 column (2.1 mm × 150 mm, 3.5 μM) using a gradient of mobile phase B (10 mM ammonium bicarbonate in 80% acetonitrile (pH 8.4)) at a flow rate of 200 μL/min (2–90%B in 30 min). For sample collection, 12 collection vials were rotated every 30 s during sample collection. The resulting 12 fractions were freeze-dried and stored at −20 °C prior to LC-MS/MS analysis..

### Fe^3+^-Immobilized Metal Affinity Purification (IMAC)

Before IMAC, 200 μg of peptides were desalted using solid phase extraction (SPE) on an HLB Oasis 1 cm^3^ cartridge (Waters). First, the cartridge was activated using 1 mL 10/90 (v/v) H2O/acetonitrile (AcN) and then equilibrated using three times 1 mL 0.1% trifluoroacetic acid (TFA). After sample loading, the cartridge was washed three times with 1 mL 0.1% TFA. Peptides were eluted in 400 μL 30/70/0.1 AcN/H_2_O/TFA and freeze-dried. A Fe(III)-NTA cartridge (Agilent) was prepared by priming it with 250 μL 0.1% TFA in AcN. The cartridge was washed three times with 250 μL 0.1% TFA in AcN. The freeze-dried eluate from the SPE was dissolved in 200 μL 30/70/0.1 (v/v/v) AcN/H_2_O/TFA and loaded on the IMAC cartridge, and the cartridge was washed three times with 250 μL 0.1% TFA in AcN. Peptides were eluted with 25 μL 1% ammonia directly into 25 μL 10% formic acid (FA), and freeze-dried.

### Dimethylation of *C. difficile* Tryptic Peptides

A tryptic digest of *C. difficile* strain 630Δ*erm* proteins was generated as described previously.^19^ Subsequently, 50 μg of tryptic peptides were diluted in 0.1% FA to a final volume of 1 mL and applied to a HLB Oasis 1 cm^3^ cartridge (Waters) which was activated using 1 mL 10/90 (v/v) H_2_O/ACN and equilibrated using three times 1 mL 0.1% formic acid (FA). After washing three times with 1 mL 0.1% FA, the dimethylation labeling mixture (4.5 mg sodium phosphate cyanoborohydride (NaCNBH_3_), 14 μL formaldehyde (CH_2_O) 37%/2.5 mL sodium phosphate buffer pH 7.5) was added for 5 min, applying 0.5 mL at 1 min-intervals of incubation.^31^ Then, the cartridge was washed three times with 0.1% FA. Finally, labeled peptides were eluted with 400 μL of an 80/20/0.1 (v/v/v) AcN/water/FA solution and freeze-dried.

### Analysis of TMT-Labeled *P. aeruginosa* Flagellin Peptides

For the data dependent analyses, the PAO1 TMT-labeled peptides were dissolved in 0.1% FA and analyzed by online C18 nanoHPLC MS/MS using an Ultimate3000nano gradient HPLC system (Thermo, Bremen, Germany) coupled with an Exploris480 mass spectrometer (Thermo). Fractions were injected onto a cartridge precolumn (300 μm × 5 mm), C18 PepMap, 5 μm, 100 A, (100/0.1 water/(FA) v/v) at a flow of 10 μL/min for 3 min (Thermo, Bremen, Germany) and eluted via a homemade analytical nano-HPLC column (30 cm × 75 μm); Reprosil-Pur C18-AQ 1.9 μm, 120 A (Dr. Maisch, Ammerbuch, Germany) at a flow of 250 nL/min. The gradient was run from 2 to 36% solvent B (20/80/0.1 water/acetonitrile/formic acid (FA) v/v/v) in 120 min. The temperature of the nano-HPLC column was set to 50 °C (Sonation GmbH, Biberach, Germany). The nano-HPLC column was drawn to a tip of ∼10 μm and acted as the electrospray needle of the MS source. The mass spectrometer was operated in data-dependent MS/MS mode for a cycle time of 3 s, with a normalized HCD collision energy of 36% and recording of the MS2 spectrum in the Orbitrap. In the master scan (MS1) the resolution was 120,000, the scan range *m*/*z* 350–1600, at a Standard AGC target with a maximum fill time of 50 ms. Dynamic exclusion after *n* = 1 with an exclusion duration of 45 s and a mass tolerance of 10 ppm. Charge states 2–5 were included. For MS2 precursors were isolated with the quadrupole with an isolation width of 1.2 Da. The first mass was set to 110 Da and the MS2 scan resolution was 30,000.

All raw data were converted to peak lists using Thermo Proteome Discoverer 2.4.1.15. and searched against the *P. aeruginosa* PAO1 database (UP000002438, downloaded from Uniprot on April 13, 2020, number of entries: 5564) using Mascot v. 2.2.07. Trypsin+chymotrypsin (C-term FKLRWY, not before P) were selected as enzyme specificity with a maximum of two missed cleavages. Mass tolerances of 10 ppm and 0.02 Da for precursor and fragment ions were used, respectively. TMTPro (K), TMTPro (N-term), and Carbamidomethyl (C) were selected as fixed modifications. Methionine oxidation, acetylation (Protein N-term) and wildtype b-type glycan (neutral mass 355.103 Da) were selected as variable modifications. Peptides with an FDR < 1% based on Percolator^32^ were accepted. Quantification of the data was performed using the reporter ion intensities of TMTpro labels to compare relative peptide abundance across samples.

The IMAC purified TMT-labeled peptides were analyzed by online C18 nanoHPLC MS/MS using an Easy nLC 1200 gradient HPLC system (Thermo, Bremen, Germany), and an Orbitrap Fusion LUMOS mass spectrometer (Thermo). The LC gradient was similar as described above. Instead of a data dependent analysis, MS/MS was performed on the predefined precursor ion at *m*/*z* 1116.318 ([M + 4H^+^]^4+^), corresponding to the tryptic + chymotryptic *P. aeruginosa* TMT-labeled flagellin peptide QVGSNGAGTVASVAGTATASGIASGTVNLVGGGQVK with two b-type modifications. The first mass was set to 110 Da and the MS2 scan resolution was 30,000. Quantification of this peptide was again performed using the reporter ion intensities of TMTpro labels.

### Mass Spectrometry of *P. aeruginosa* ProtK Flagellin Peptides and Methylated Type A-Modified Tryptic Flagellin Peptides from *C. difficile*

For the MS analysis of IMAC purified *P. aeruginosa* ProtK peptides, samples were dissolved in 0.1% FA and analyzed by online C18 nanoHPLC MS/MS using an an Easy nLC 1200 gradient HPLC system (Thermo, Bremen, Germany), and an Orbitrap Fusion LUMOS mass spectrometer (Thermo). Fractions were injected onto a homemade precolumn (100 μm × 15 mm; Reprosil-Pur C18-AQ 3 μm, Dr Maisch, Ammerbuch, Germany) and eluted via a homemade analytical nano-HPLC column (30 cm × 75 μm; Reprosil-Pur C18-AQ 1.9 μm). The analytical column temperature was maintained at 50 °C with a PRSO-V2 column oven (Sonation, Biberach, Germany). The gradient was run from 2 to 40% solvent B (20/80/0.1 water/acetonitrile/formic acid (FA) v/v) in 120 min. The nano-HPLC column was drawn to a tip of ∼5 μm and acted as the electrospray needle of the MS source. The MS spectrum was recorded in the Orbitrap (resolution 120,000; *m*/*z* range 400–1500; maximum injection time 50 ms). MS/MS was performed using HCD at an NCE of 30%. Dynamic exclusion was after *n* = 1 with an exclusion duration of 15 s with a mass tolerance of 10 ppm. Charge states 1–5 were included.

For MS/MS(/MS) analysis, samples were reanalyzed on the above system using a shorter gradient (2–40%B in 60 min). Fragmentation (HCD, NCE 30%) was performed on the predefined precursor ion at *m*/*z* 737.797 ([M + 2H^+^]^2+^), corresponding to the flagellin peptide TASGIASGT with two b-type glycans. For the subsequent MS3 experiments (HCD, NCE 30%), fragment ions at *m*/*z* 356.110 and 130.086 were selected. The same MS/MS(/MS) method was used for the methylated Type A-modified tryptic peptides from *C. difficile* flagellin with predefined precursor and fragment ions at *m/*z 751.871 and 130.086, respectively.

## Data Availability

The mass spectrometry proteomics data have been deposited to the ProteomeXchange Consortium via the PRIDE^33^ partner repository with the data set identifier PXD056085.

## Supporting Information Available

Flagellin glycosylation biosynthetic gene clusters in *P. aeruginosa* PAO1 and *Clostridioides difficile* strain 630Δ*erm* (Figure S1); MS3 fragmentation of the Type A-specific fragment ion at *m*/*z* 356.110 (Figure S2); relative quantification of PA1088-PA1091 in the mutant strains (Figure S3); relative quantification of FgtA (PA1091) peptides in the pa1091 mutant strain based on a quantitative proteomics (Figure S4); MS/MS spectra of the flagellin tryptic+chymotryptic peptide QVGSNGAGTVASVAGTATASGIASGTVNLVGGGQVK with two deoxyhexoses or nonglycosylated (Figure S5); overview of the *P. aeruginosa* PAO1 strains used in this study (Table S1); overview of the TMTpro labels per strain (Table S2) (PDF)

## Abbreviations

TMT: Tandem Mass Tagging
IMAC: Immobilized Metal Affinity Chromatography
AcN: acetonitrile
FA: formic acid
Thr: threonine
pThr: phospho-threonine
Me: *N*-methyl
diMe: *N,N*-dimethyl
LB: Luria–Bertani
MS3: MS/MS/MS
CID: collisional-induced dissociation
HCD: higher energy collision dissociation
Q-TOF: quadrupole time-of-flight
FDR: false discovery rate
SPE: solid phase extraction
