# Supplementary material for: Elucidation of the Glycan Structure of the b-type Flagellin of Pseudomonas aeruginosa PAO1
Source: ACS Infect Dis. 2025 Jan 24;11(2):518–28. doi: 10.1021/acsinfecdis.4c00896 (PMC11833859; doi:10.1021/acsinfecdis.4c00896)
Supplement: Supplementary file 1 — id4c00896_si_001.pdf [file id4c00896_si_001.pdf]

Supporting information

Elucidation of the glycan structure of the b-type flagellin of *Pseudomonas aeruginosa* PAO1

Paul J. Hensbergen[1*], Loes van Huijkelom[1], Jordy van Angeren[1], Arnoud H. de Ru[1], Bart Claushuis[1], Peter A. van Veelen[1], Wiep Klaas Smits[2], Jeroen Corver[2]

[1] Center for Proteomics and Metabolomics, Leiden University Medical Center, Leiden, 2333 ZA, The Netherlands

[2] Leiden University Center for Infectious Diseases, Leiden University Medical Center, Leiden, 2333 ZA, The Netherlands

*Correspondence to:

P.J. Hensbergen

Center for Proteomics and Metabolomics

Leiden University Medical Center

PO Box 9600

2300 RC Leiden

The Netherlands

Tel.: +31-71-5266394

E-mail: P.J.Hensbergen@lumc.nl

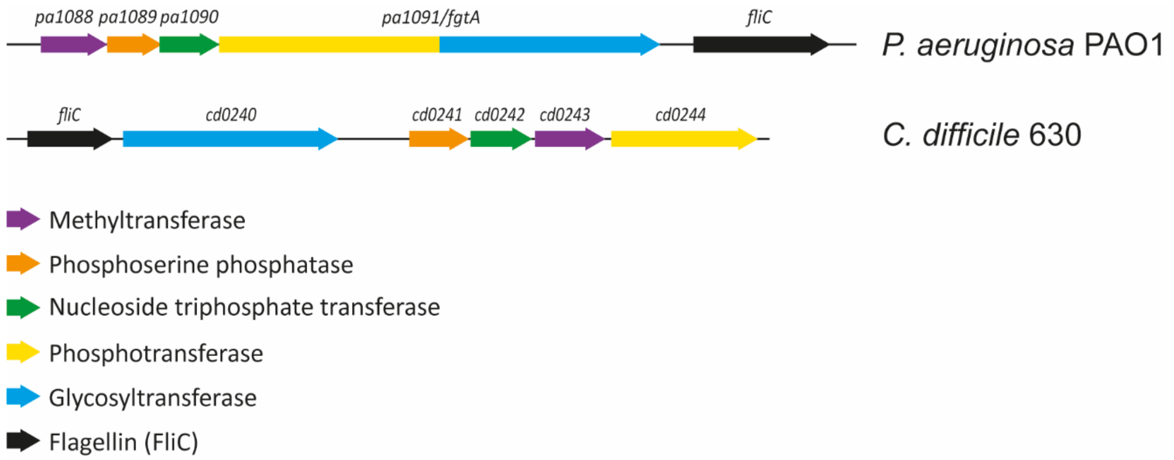

Supplemental Figure S1: **Flagellin glycosylation biosynthetic gene clusters in *Pseudomonas aeruginosa* PAO1 and *Clostridioides difficile* strain 630Δ*erm*.**

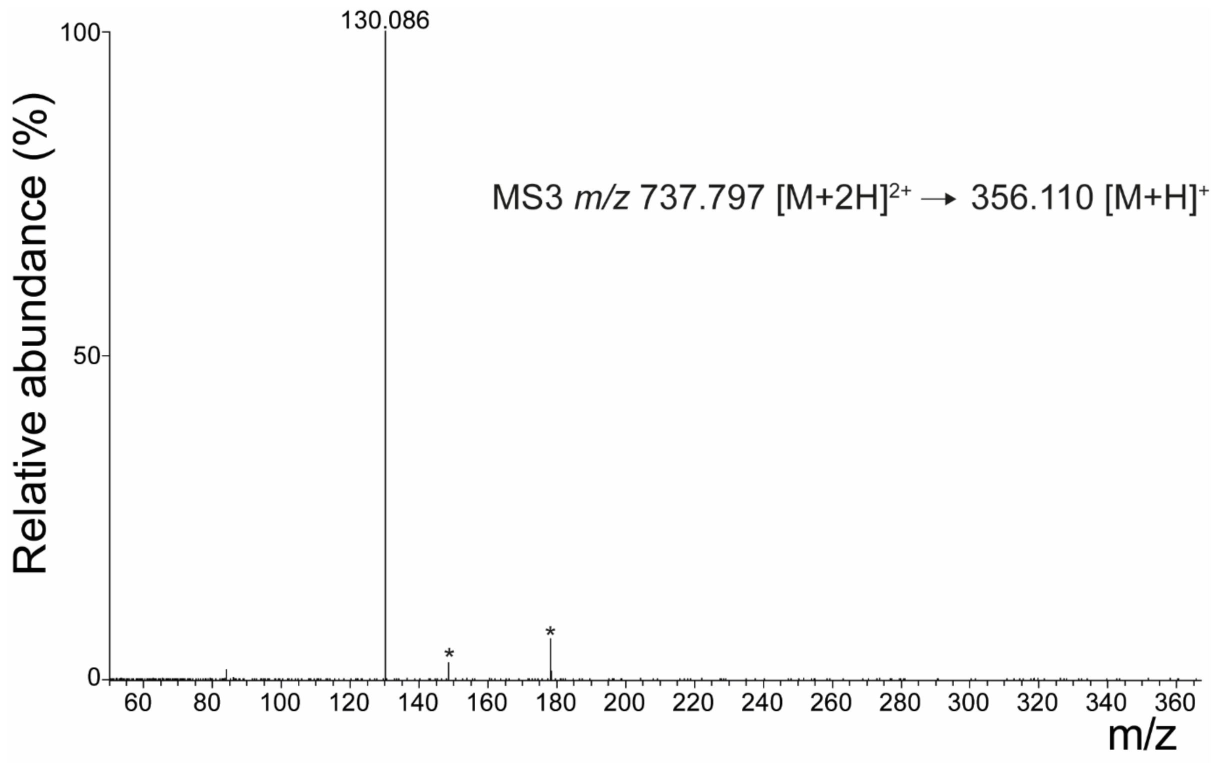

Supplemental Figure S2: **MS3 fragmentation of the Type A-specific fragment ion at *m/z* 356.110 as observed in Fig. 3A**. The signals indicated with an asterisk (*) are background signals of unknown origin that were observed in all fragmentation spectra.

Gene	Protein	Uniprot ID	Coverage (%)	#peptides	#PSMs	#quant peptides
pa1088	PA1088	Q9I4P1	13	4	4	4
pa1089	PA1089	Q9I4P0	27	4	4	2
pa1090	PA1090	Q9I4N9	16	2	2	1
pa1091	FgtA (PA1091)	Q9I4N8	23	36	42	21

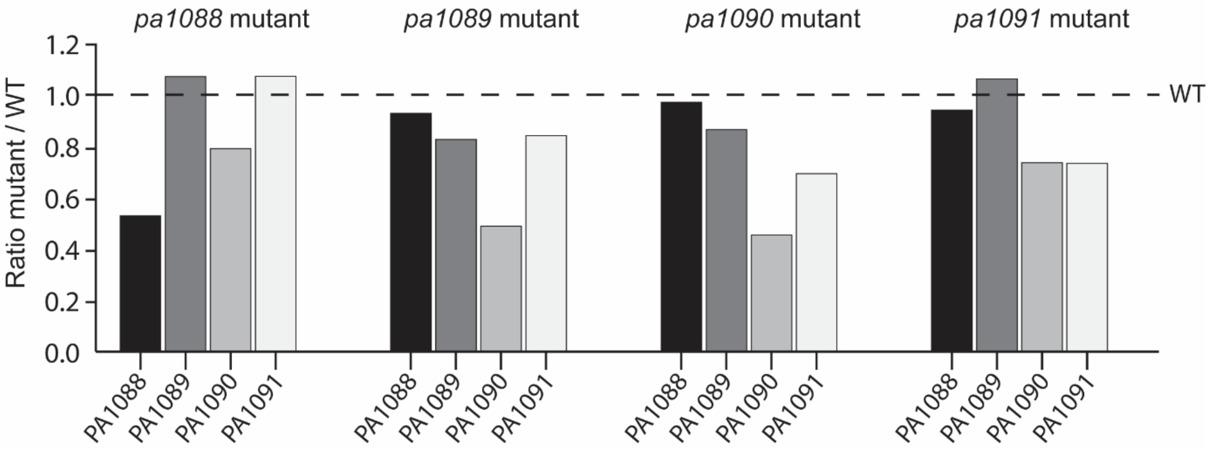

Supplemental Figure S3: **Relative quantification of PA1088-PA1091 in the mutant strains.**

```
>tr|Q9I4N8|Q9I4N8_PSEAE Flagellar glycosyl transferase, FgtA OS=Pseudomonas
aeruginosa (strain ATCC 15692 / DSM 22644 / CIP 104116 / JCM 14847 / LMG
12228 / 1C / PRS 101 / PAO1) OX=208964 GN=fgtA PE=4 SV=1
MIEDSVSEPDRRDGGDAGKRLAWLQRQVLPVLEEGGAVWVSGLDGAPFAGGAHKVVEASV
AGELPGNERFRLACLGGAVGGVADDWQAMRLLLRAVESLEDRGWLLLEEALSVSAAGACR
SPQAQARLALSLGLRQVAELRLGAPDDGGRQRVLQLFRQDLAVARMQRYSGLRVACYGNM
PFHYRSLRPLAECFEDSLLSLDIDEVMAWKPDVIAVADGWSVEFWRDYCDAHNVLLVGMR
HGSVTRYGFAEGTYRYADYLCGSAWDIDDTLASSVMPRNGFLLTGNAWCDEVFRLPARTP
AENAPTILFAPTYNPEISAAVHLGERVVALIRKVYPASRIIIKPHPAIVQHEHAFVSDKD
LFRDLMKLWREQSRTDPLVTLVDDPEASIAASFAEADILLADRSSLIFEFMTLDRPILLF
SREQRIARWAYNPEAPGNAWRDIGLEFADDEQLLDLLANAFTRHAESRDTQENRTQQLYG
RFRDGRSVQRVAAAIAEAPRLQIVLDARAAADGGQRLAEHYAACFAHRRIDLIAPSGAAL
PADVRRFDSWRAWCDAEAATLERRASAVLLVEDDGQLLPGSAHQVSALLPAIAMGQLRHA
RLALEEREAGPAGETDWSARRLHRLLARLQPKPAWRLLAPALLREELADLPDEAEQPLWL
RAVAAAEAPVEWPVGQLNLFAEEGLLRQGDVHMLCARARLRAVPCVSGLPLRQRQVVLQV
TPFASQLYDRFPFQLRVCLNGQLFSRVSVEDARAKSLVLPFRGDERGGMTIELECDGSYP
AFEGVSTPVSLLLQTAFQPAPGDYCAALAAGAASPEAGEDLRSEYETWLHGREVEPARYR
PFIDALRRDSRIEVLVLAEEAEDADLQRSLASIDGQALPAWRTRILGRAPAFARKGLAWL
AEGGSAAERINLAAAASDADWLIVIHAGDELARSALLLLAEKIRTETALLCCYSDEDHVC
DGRYEAPLLKPDFNLDLLRSYPYCGRSLAFQRAALLAQGGLQEGFGDLALQDFMFRLAER
EGLDRIGHLAEVLYHSARAFGEWLASAAVRPFIASVVDEHLNRLGVPHRIEPGRLAVINR
IAYDYPGTPAVSLLLPVGDSLSALQRSVESFLENTDYPSYELLLVASGPLAPDMAAWLEA
VQGLGSEQIRVLSPQASSLAGCLNLCVAEARGEFLLSLGAGVVALRPDWLRELLNHGRRP
EVGAVGGKLLGLDGTIREAGLVLGLGGTAGRAFAGEAGDSAGYMHRLLVVQNHTALSASC
LLFKRSLHDELGGFDENDFAHGHADVDFSLRARQLGYLSVWTPYAILAQNGNVELPSVEA
DESLYRRWLPALARDPAYNRNLSLEGAGFALERPEVPAWQPLFGRSPLPRVLAHPADPYG
CGHYRVRQPFRALHDAGLLDGMLSESLLQPVALERLEVDSVILQRQISEEQLRAISRMRS
FNRAFRVYELDDYLPELPLKSLHRAEMPGDIRQILGRALGLADRFVVSTEPLAEAFRRMH
GDIRVVPNRLPLPWWRDLSSRRRDAERPRVGWAGGIGHGGDLEVIAEVVRELADEVDWVF
FGFCPDALRPYVREYHPGVEIERYPAYLASLDLDLALAPLEQNRFNECKSNLRLLEYGVL
GFPVICSDVLCYRDSLPVTRVKNRSRDWLEAIRAHLADADANAAAGAQLREAVRRDWMLE
GAHLEAWAAAWLPD
```

Supplemental Figure S4: **Relative quantification of FgtA (PA1091) peptides in the pa1091 mutant strain based on a quantitative proteomics experiment using TMT.** Peptides colored in red had a ratio of pa1091 mutant/WT below 0.7 (0.27-0.65, median 0.50, n=8). Peptides colored in yellow had a ratio of pa1091 mutant/WT above 0.7 (0.78-1.39, median 0.92, n=14).

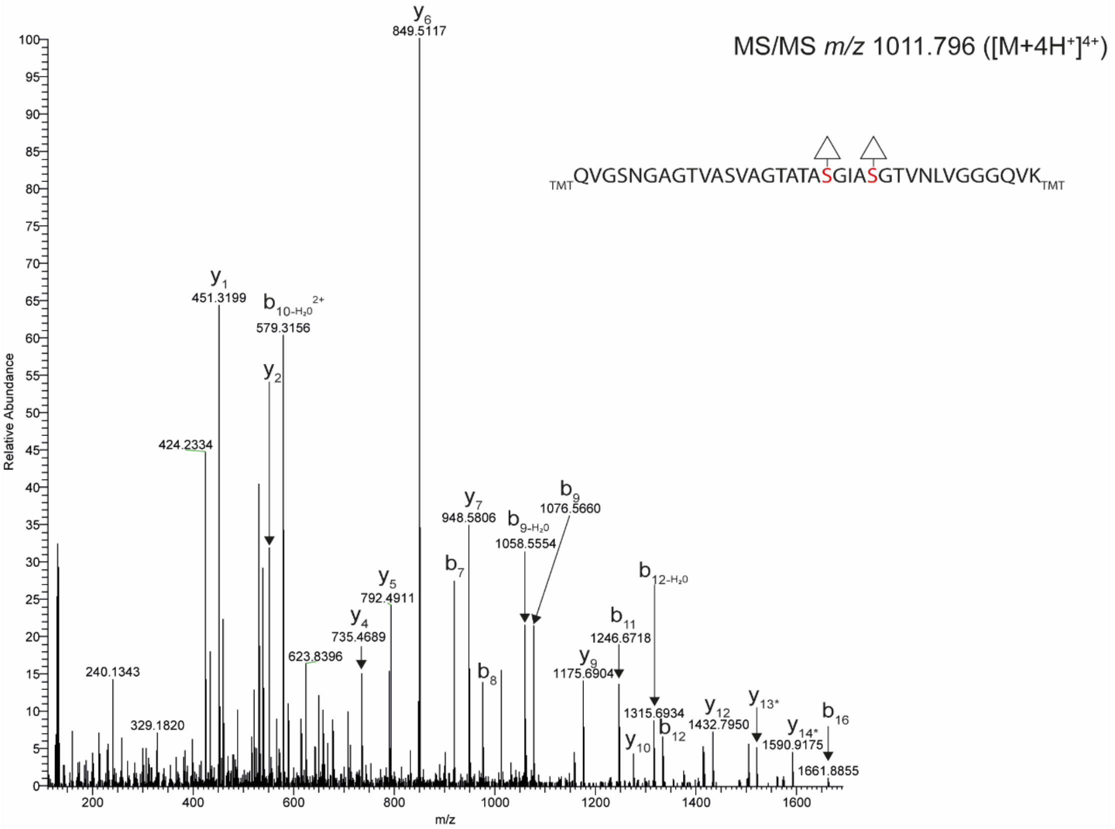

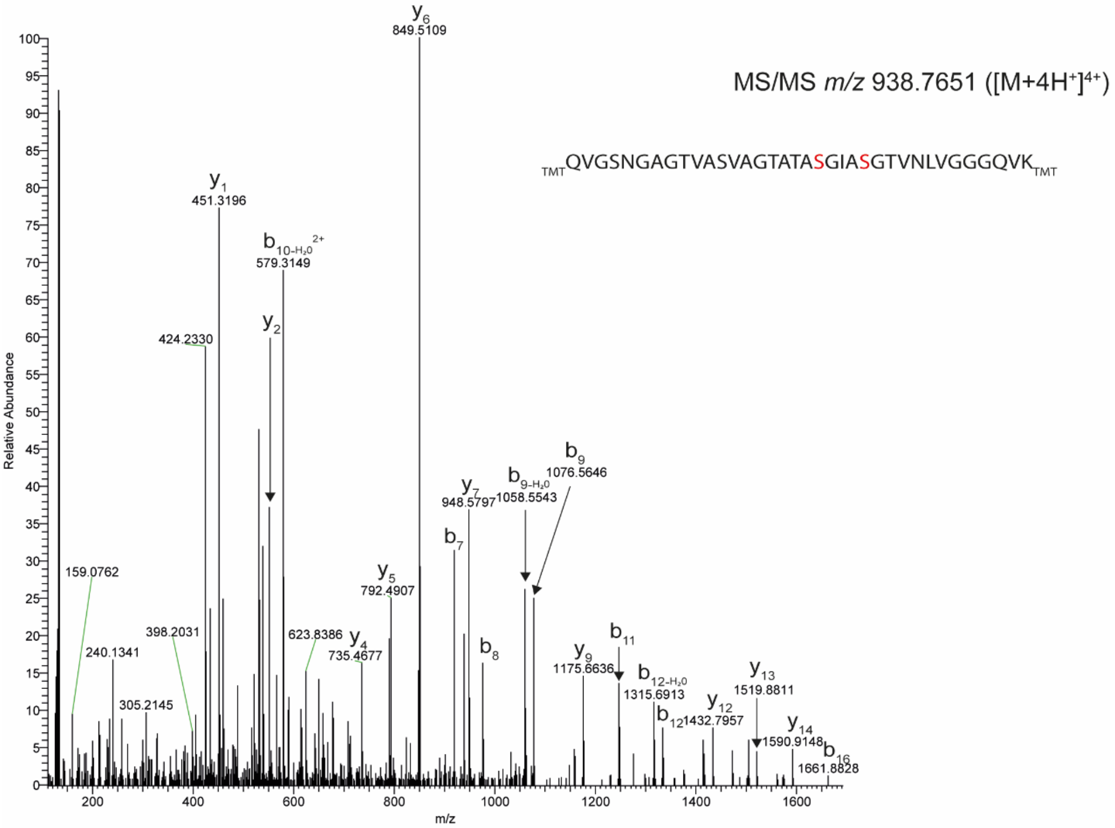

Supplemental Figure S5: **MS/MS spectra of the TMT-labeled flagellin tryptic+chymotryptic peptide QVGSNGAGTVASVAGTATASGIASGTVNLVGGGQVK with two deoxyhexoses (upper panel) or non-glycosylated (lower panel)**. *: Fragment has lost the deoxyhexose.

Supplemental Table S1: Overview of the *P. aeruginosa* PAO1 strains used in this study. *For more information about the transposon mutants see Jacobs et al. 2003, PNAS 100:14339 and Held et al. 2012, J. Bacteriol. 194:6387. *: strain ID from the Salipante lab (https://sites.google.com/uw.edu/salipante-lab).*

Strain	Original strain ID*	Genotype	Locus tag	Description
BC107	n.a.	WT PAO1	n.a.	WT
BC108	PW2962	PA1088-C10::ISLacZ/hah	PA1088	*pa1088* mutant
BC111	PW2965	PA1089-F11::ISphoA/hah	PA1089	*pa1089* mutant
BC112	PW2967	PA1090-A07::ISphoA/hah	PA1090	*pa1090* mutant
BC115	PW2969	PA1091-A02::ISphoA/hah	PA1091	*pa1091* mutant

Supplemental Table S2. Overview of the TMTpro labels per strain.

**TMTpro label**	**Strain**	**Genotype**	**Description**
126	BC107	WT	WT
127N	BC107	WT	WT
127C	BC107	WT	WT
128N	BC108	PA1088-E05::ISLacZ/hah	*pa1088* mutant
128C	BC108	PA1088-E05::ISLacZ/hah	*pa1088* mutant
129N	BC108	PA1088-E05::ISLacZ/hah	*pa1088* mutant
129C	BC111	PA1089-B10::ISphoA/hah	*pa1089* mutant
130N	BC111	PA1089-B10::ISphoA/hah	*pa1089* mutant
130C	BC111	PA1089-B10::ISphoA/hah	*pa1089* mutant
131N	BC112	PA1090-B10::ISphoA/hah	*pa1090* mutant
131C	BC112	PA1090-B10::ISphoA/hah	*pa1090* mutant
132N	BC112	PA1090-B10::ISphoA/hah	*pa1090* mutant
132C	BC115	PA1091-B10::ISphoA/hah	*pa1091* mutant
133N	BC115	PA1091-B10::ISphoA/hah	*pa1091* mutant
133C	BC115	PA1091-B10::ISphoA/hah	*pa1091* mutant
